# Micromotors Meet Collective (Bio)sensing: The Asset Behind the Assay

**DOI:** 10.1021/acs.analchem.5c00619

**Published:** 2025-06-19

**Authors:** Alberto Escarpa, Beatriz Jurado-Sánchez

**Affiliations:** † Department of Analytical Chemistry, Physical Chemistry, and Chemical Engineering, Universidad de Alcala, Alcala de Henares, E-28802 Madrid, Spain; ‡ Chemical Research Institute “Andres M. del Rio”, Universidad de Alcala, E-28802 Madrid, Spain

## Abstract

Micromotors are microscale devices with enormous potential for analytical (bio)­sensing due to autonomous motion capabilities in extremely small sample volumes or for guided detection in localized hard-to-reach areas. These unique features enable dynamic interactions with the analytes, offering considerable promise in microscale environments and opening new avenues for on-the-fly (bio)­sensing strategies. By selecting and discussing the ideas and findings behind pioneering works, we offer our perspective on the current state of the art in the field of *in vitro* (bio)­sensing approaches based on the micromotors classified according to their detection principle: motion-based, optical, and electrochemical sensing. We will also draw attention to current challenges and opportunities that have not yet been fully explored, in a landscape that is as exciting as it is changing.

## Graphical abstract

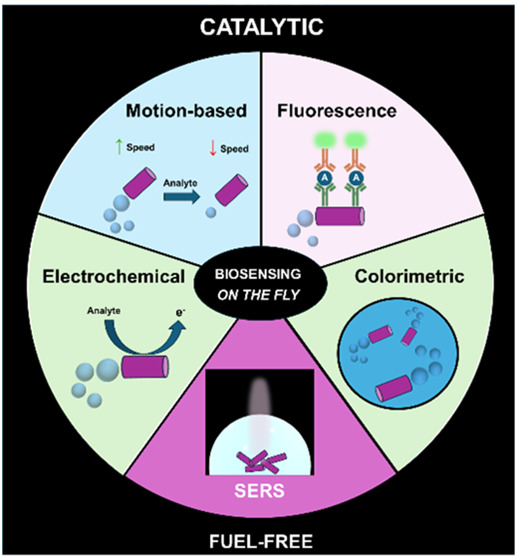

## Why Micromotors form (Bio)sensing?

Micromotors are particles ranging in size from a few nanometers to about 200 μm that have the unique ability to move autonomously in solution.^1^ Recently the history of micromotors was reviewed.^2^ The field began with the report of the self-electrophoretic motion of nanorod-based micromotors in 2004.^3^ Since then, the field has experienced a vast development, with many applications in the environmental, biomedical, and analytical field, the latter of which is much less explored.^4,5^ We will mainly refer to this field from this perspective.

Proper detection of biomarkers is essential for diagnosing illnesses, but this remains challenging in biological and clinical samples with limited availability, which hinders reliable detection. The ultrasmall dimensions of micromotors, along with their accessibility and force, open up new possibilities for sensitive and selective detection in these small sample volumes. The continuous, autonomous movement of micromotors through low sample volumes and the modulation of mass transport by the self-vortex effect resulting from their locomotion significantly enhance interactions with the target analytes. The autonomous movement of micromotors accelerates the reaction kinetics, enabling fast detection of the analyte, even in viscous or raw samples without prior treatment.^6–8^ Another major potential of micromotors in (bio)­sensing is their ability to perform precision-guided targeted (bio)­sensing in perfectly localized, well-defined, and/or difficult-to-reach areas, opening up enormous possibilities in precision (bio)­sensing.

Interestingly, when trying to answer the disturbing question of why micromotors are used for (bio)­sensing, one must consider a unique feature of this technology: their collective behavior. This unique feature places micromotors at the forefront of the biosensing field, where the required detection of a few molecules present in extremely low sample volumes, the omnipresent challenge of achieving sensitivity at the microscale, will only be possible due to the swarm of micromotors, which are convectively and collectively driven, even for guided detection in localized and well-defined hard-to-reach localized areas.

In this niche, micromotors offer a unique solution to overcome such challenges, providing a plethora of novel on-the-fly (bio)­sensing approaches and opening up new and exciting opportunities that are still being explored and are limited only by our imagination.

Micromotors can be classified into three main groups according to their propulsion mechanism: (a) (bio)­chemically powered (catalytic) motors;^9^ (b) fuel-free micromotors powered by biocompatible sources, such as magnetic, ultrasound, light, electric fields, and thermal energy;^10^ and (c) systems “towed” to the site of interest by living organisms, such as cells and bacteria.^11^

To date, the different micromotor-based (bio)­sensing approaches can broadly be classified according to the *in vitro* detection principle: (i) motion-based sensing, monitoring changes in micromotor speed due to the action of certain analytes; (ii) optical and (iii) electrochemical sensing. From this perspective, we will provide a realistic state of the art on the progress, the challenges faced, and the opportunities envisaged for micromotors in the field of *in vitro* (bio)­analytical sensing according to these three types of detection principle, following the chronological roadmap shown in Figure 1.

**Figure 1:**
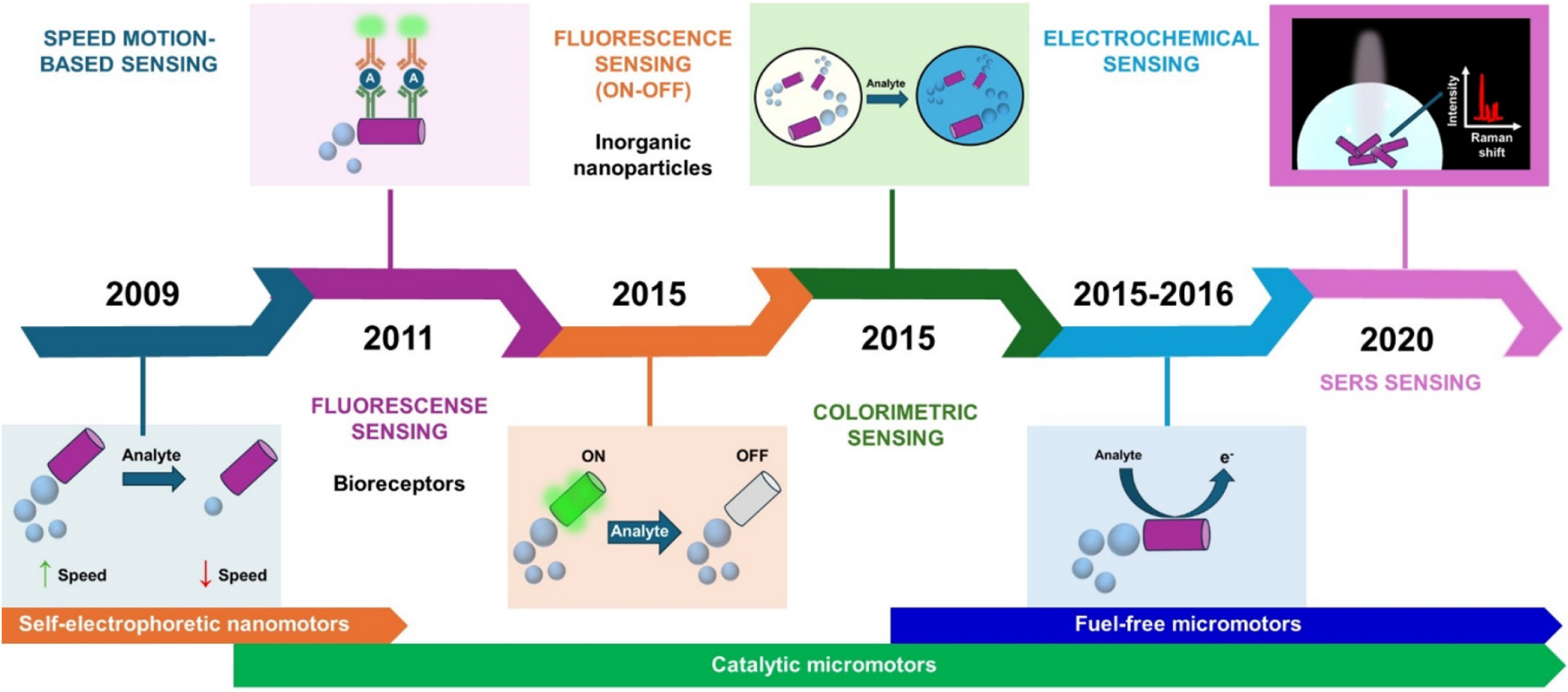
A chronological roadmap of (bio)­sensing approaches based on micromotors with an estimated date of when the sensing principle was first used. Motion-based sensing is based on the variation in the micromotor speed induced by the analyte concentration. Optical sensing is based on micromotors triggering the generation of fluorescence or colorimetric signals, which can be related to the analyte concentration. In the first format, micromotors are modified with a specific bioreceptor (e.g., an antibody for the analyte, A), followed by the interaction with a secondary antibody labeled with a fluorescent tag, which increases the fluorescence on the micromotor surface. In the second format, the micromotors are modified with fluorescent inorganic nanoparticles that are quenched by the action of the target analytes. Colorimetric sensing relies on generating colored solutions in the presence of the analyte through chemical reactions. Electrochemical sensing relies on generating an electrochemical signal coupled with a micromotor event, using both unmodified and biofunctionalized micromotors with receptor probes that can be monitored using miniaturized electrodes. Surface-enhanced Raman scattering (SERS) sensing uses micromotors as on-the-fly substrates.

## Micromotor Motion-Based (Bio)sensing

Motion-based sensing has been explored since the early days of micromotor technology as this detection principle is inherent to micromotors due to their self-propulsion. The first study to demonstrate the potential of self-propelled micromotors for sensing was reported by Wang’s group in 2009, following the observation of greatly accelerated motion of Au–Pt nanowire motors in the presence of silver ions.^12^ However, this strategy had a key limitation: the lack of selectivity, as other ions could interfere with the motion of the nanowires. To solve this challenge, the concept was extended using Ag-tagged DNA receptors. Upon hybridization with the target DNA, the silver was dissolved in a hydrogen peroxide solution, thereby increasing the micromotor’s speed in a turn-on format.^13^ Self-electrophoretic propulsion mechanisms used for nanowire motion are also greatly affected by the presence of electrolytes in solutions, which prevents their practical application beyond ultrapure water. Nevertheless, these pioneering designs have inspired analytical chemists to explore the enormous potential of micromotors for analytical sensing, mainly due to their ability to operate in extremely low sample volumes, which is not possible with traditional static biosensors.

The introduction of bubble-propelled catalytic micromotors in 2011 represented a convenient solution to this challenge, as their motion is not greatly affected by the presence of electrolytes in the solution due to their high towing force.^14–17^ In this approach, oxygen bubbles are typically generated on Pt catalytic layers using hydrogen peroxide as a fuel. This was undoubtedly a milestone reached and opened the door to micromotor-based biosensing in extremely low sample volumes, as illustrated in Figure 1, which shows the widespread use of the catalytic approach in (bio)­sensing from its introduction to the present day.

The poisoning of enzyme (catalase–urease) propelled micromotors and the subsequent motion-based reduction were explored for the detection of heavy metals or nerve agents.^18–20^ Pt-based microtubes can also be poisoned by sulfur-containing compounds, as well as Pb and Cd.^21,22^ Such species can bond to Pt, decreasing its catalytic activity.^23^ However, the lack of selectivity is not the only drawback; high-resolution optical microscopes are also required to track and monitor changes in micromotor speed reproducibly, which is of paramount significance for analytical purposes. This explains why motion-based approaches have rarely been explored, although with distinctive developments to date.

One of the main ways to expand the potential of motion-based approaches is to increase selectivity and integrate the strategies into microscope-free, portable, point-of-care devices. These devices could be used by nonspecialist users in clinical practice, given that micromotor-based approaches only require microliters. For example, a micromotor device based on smartphones has been proposed for detecting the HIV-1 virus. This device incorporates a loop-mediated isothermal amplification module for virus amplification. Subsequently, the amplified solution is mixed with DNA-modified polystyrene (PS)/Pt catalytic micromotors. If the HIV-1 virus is present, specific hybridization results in assembly with Pt-containing micromotors for turn-on sensing. Other existing micromotor motion-based integrated detection strategies are illustrated in Figure 2. Like HIV-1 detection, the ZIKA virus has been determined using a portable smartphone in a turn-on format. This strategy is depicted in Figure 2A. The smartphone device incorporates a microchip for minimal sample treatment and detection. In the first step, the sample containing the analyte/virus is mixed with anti-Zika monoclonal antibody-modified polystyrene beads. The micromotors consisted of 20 nm Pt nanoparticles modified with the anti-Zika antibody. If the virus is present, the Pt nanomotors assemble with the complex to achieve a highly accelerated speed due to an increase in the number of Pt nanoparticles. The smartphone device can record videos and automatically analyze the motion of the beads. The device was used to measure real infected samples from patients with 100% efficiency in classifying them as positive or negative. However, quantitative analysis was not performed.^24^

**Figure 2:**
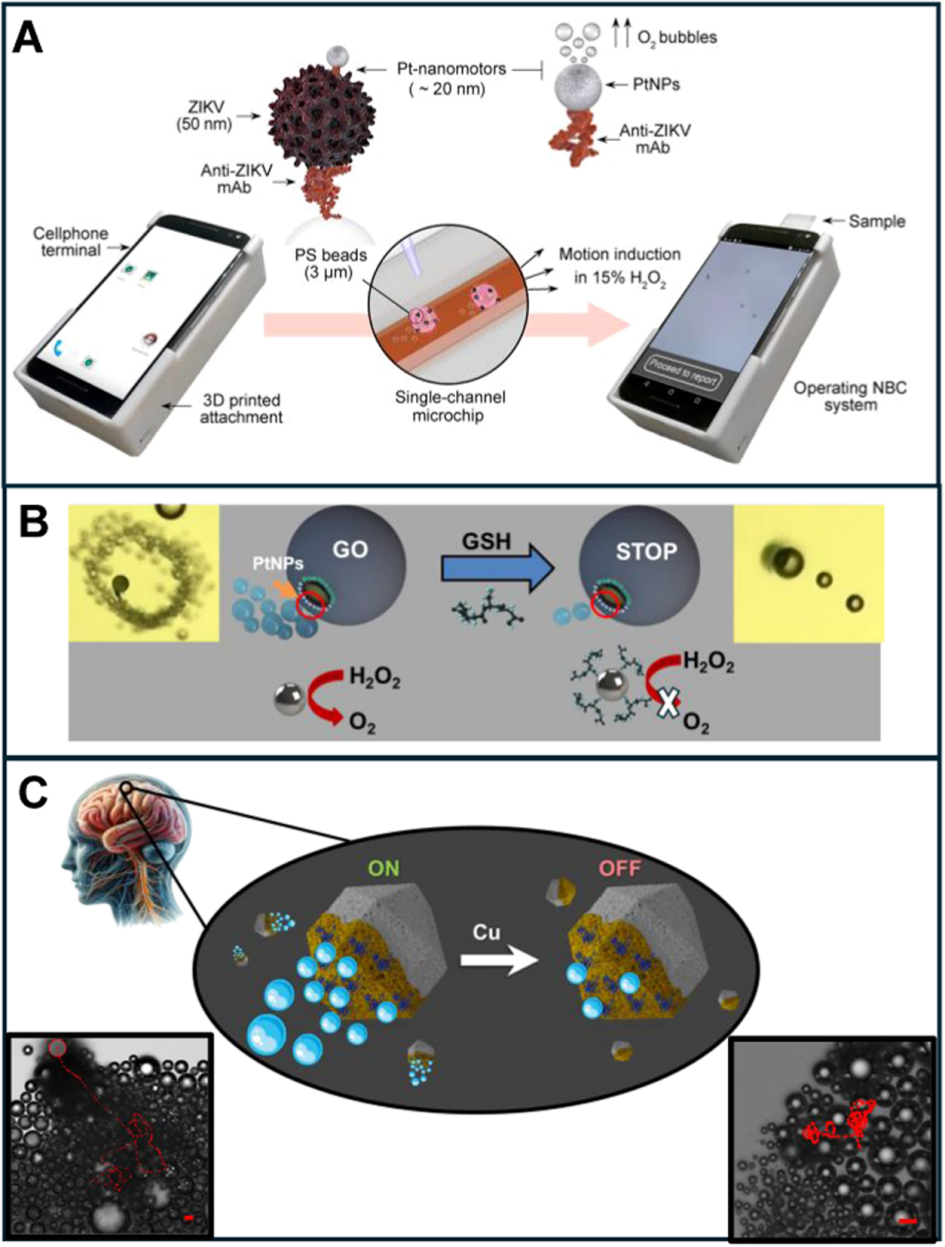
Micromotor motion-based (bio)­sensing approaches. (A) Turn-on sensing of ZIKA virus. (B) Turn-off sensing of glutathione (GSH). (C) Turn-off sensing of copper in cerebrospinal fluid samples with zeolitic imidazole (ZIF)-8 MOFs/gold/catalase micromotors. Reprinted from ref 24 (A) ref 25 (B) and ref 26 (C) copyright 2024 Royal Society of Chemistry.

A more simplified approach involved equipping a 3D-printed device integrating a mobile phone with a low-cost, high-resolution optical lens to observe the motion of micromotors, as shown in Figure 2B. To illustrate the concept, motion-based sensing of glutathione was demonstrated by poisoning the Pt catalytic layer of graphene oxide-based micromotors with the thiol groups present in glutathione. The device can accommodate any type of smartphone equipped with a universal, high-resolution optical lens. The sample can be placed directly on a glass slide on a tailored platform for focusing, such as an optical microscope. Excellent correlation match was obtained using both the portable, smartphone-based device and a high-resolution optical microscope, demonstrating the enormous potential of micromotors in microscope-free devices. However, due to the inherent difficulty of accurately measuring velocity changes in real media, only fortified samples were analyzed.^25^

A recent example of using micromotor speed as an analytical signal is the detection of copper (Cu) in cerebrospinal fluid samples from the patients with Alzheimer’s disease using smart biocatalytic surfaces from metal–organic frameworks (MOFs). Catalase activity decreased with Cu poisoning, resulting in a concentration-dependent decrease in speed.^26^ While promising, this strategy could lack exquisite selectivity due to the presence of other metals, amyloid plaques, proteins, and sample constituents that can influence motion. However, considering the low availability and the invasive nature of sample collection, fast screening tools can be beneficial for prompt treatment and diagnosis of severe illnesses such as Alzheimer’s disease. Indeed, the presence of essential metals, such as Zn, does not interfere with the antagonistic role of Cu, and the screening results allow for differentiation between groups of healthy individuals and those in the early stages of Alzheimer’s disease from those in more advanced stages. It should be noted that diagnosing Alzheimer’s disease is complex, and detecting its biomarkers is difficult due to the low volume of available clinical samples. Thus, it is important to recognize the enormous potential of new technologies, such as the new generation of smart micromotor-based on MOFs, in clinical diagnostic screening.

Although speed-based detection is an inherent and elegant detection principle for micromotors in (bio)­sensing due to transduction simplicity and selective functionalization toward biomarkers, the analytical signal is highly dependent on the medium’s properties (e.g., viscosity) and subject to higher variability, which complicates analytical quantification. These drawbacks can be overcome with suitable calibration approaches and by measuring many significant trajectory traces to obtain reliable speeds in collective behavioral motion. Further efforts are needed, requiring cross-sectional collaborations with engineers to develop dedicated algorithms that provide a direct YES/NO response on smartphones, thereby extending their use to nonspecialized personnel.

## Micromotor-Based Optical (Bio)sensing

Following the roadmap in Figure 1, optical sensing approaches represent the next logical step in developing micromotors for analytical (bio)­sensing applications. In this section, we will first discuss the approaches using fluorescence, followed by visual/colorimetric detection, and finally, SERS.

### Micromotor-Based Fluorescence (Bio)­sensing

This detection approach has been implemented using two types of fluorescent tags: inorganic nanoparticles with inherent fluorescence, such as quantum dots (QDs); and bioreceptors with fluorescent tags, such as antibodies, aptamers, and affinity peptides. Although early works were done using antibodies, as shown in Figure 1, we have chosen to first discuss the approaches based on inorganic particles, due to the further development of assays based on molecular recognition assays using bioreceptors. Additionally, we will discuss some studies that use fluorescent analytes retained in molecularly imprinted sites in micromotors at the end. Figure 3 shows representative examples of the work to be discussed.

**Figure 3:**
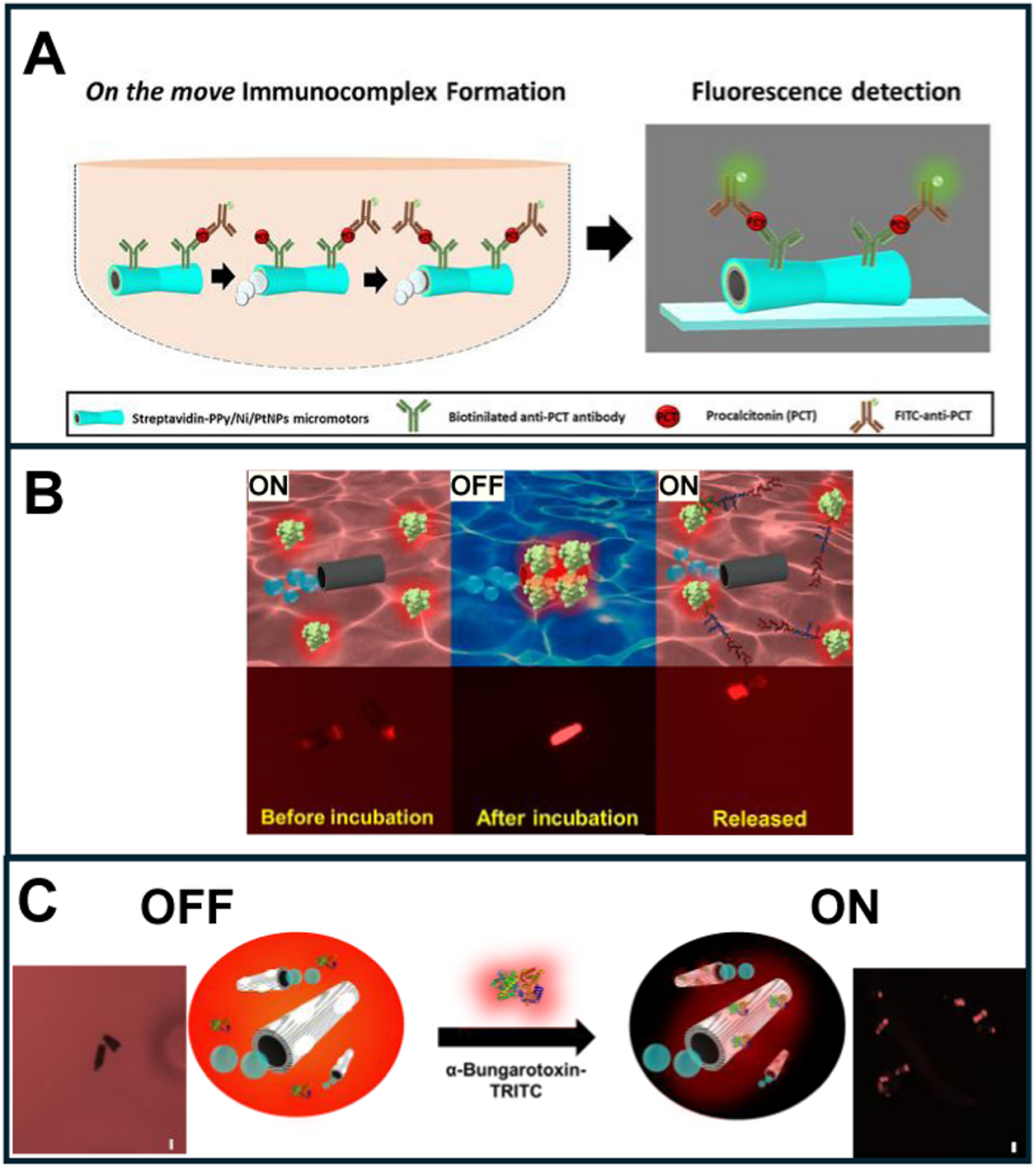
Fluorescence (bio)­sensing using micromotors. (A) Polymer-based catalytic micromotors modified with antibodies for fluorescence detection of PCT. The approach relies on the modification with a specific antibody, dynamic capture and labeling with a FITC-labeled secondary antibody for fluorescence detection. (B) WS_2_/Pt catalytic micromotors modified with a rhodamine-labeled affinity peptide for bacteria endotoxin detection. (C) PEDOT MIMs micromotors for venom toxin detection using rhodamine-labeled α-bungarotoxin as the target analytes. Reprinted from ref 27 (A), ref 28 (B), and ref 29 (C).

Regarding the combination of micromotors with inorganic fluorescent nanoparticles, catalytic micromotors include PEDOT/Pt micromotors modified with CdS QDs for ON–OFF mercury detection,^30^ polycaprolactone/Pt Janus micromotors, encapsulating graphene QDs modified with aminophenylboronic acid for bacteria-related endotoxin detection,^31,32^ and rolled-up PVP/Pt catalytic micromotors or polycaprolactone micromotors, encapsulating fluorescent covalent organic frameworks for 2,4,6-trinitrophenol detection.^33,34^ The detection principle relies on the specific attachment of the target analytes to inorganic sensing probes, which changes the emission bandgap after analyte interaction and quenches the fluorescence in a concentration-dependent manner. Although speciation analysis has been demonstrated, e.g., the specific quenching on the CdS-based micromotors in the presence of Hg but not methyl-Hg,^30^ the lack of selectivity is a significant limitation of these approaches. To improve biocompatibility, the field has evolved to include fuel-free (noncatalytic) configurations. Magnetically propelled ZIF-8 micromotors have been used for the simultaneous detection and removal of uranium. These micromotors contain Fe_3_O_4_ nanoparticles as magnetic engines and poly­(acrylic acid-*co*-acrylamide) as fluorescence probes. The presence of uranium results in fluorescence quenching in a concentration-dependent manner.^35^ Yet, these works served as the basis for the development of strategies that incorporate probes to enhance analytical performance, such as antibodies and, more recently, aptamers and affinity peptides, which have exquisite selectivity and sensitivity.

Regarding antibodies, fluorescence-polymer-based catalytic micromotor immunoassays have been proposed for determining procalcitonin (PCT) as a gold standard biomarker in plasma samples (25 μL) from low-weight neonates suspected of having sepsis at clinically relevant concentrations (0.5–150 ng/mL). These results show excellent agreement with the gold standard immunoassay method used by the hospital.^27^ For more details on the strategy, please see Figure 3A. Instead of covalent functionalization, when using aptamers or affinity peptides, the assay’s principle relies on fluorescent probe attachment to the nanomaterial via hydrophobic or π–π interactions (OFF state). The analyte’s higher affinity for the bioreceptor, which incorporates the fluorescent detection probe, results in release from the micromotor surface in a concentration-dependent manner with high selectivity and sensitivity (ON state).

The potential of aptamers has been demonstrated for the detection of ricin,^36^ mycotoxins,^37^ as well as for interleukin-6 (IL-6)^38^ and PCT,^39^ in low volume samples from neonates using aptamer-modified graphene micromotors, which allow even for dual assays.^40^ A cutting-edge micromotor dual OFF–ON aptassay has been reported for the simultaneous determination of both early sepsis biomarkers, PCT and IL-6, in 15 min using only 2 μL of the sample from low-birthweight neonates with a gestational age of less than 32 weeks and birth weight below 1000 g, with clinical suspicion of late-onset sepsis. This approach achieved the high sensitivity required in clinical scenarios (pg for IL-6 and ng for PCT), demonstrating excellent reliability compared to the hospital method for both biomarkers during analysis of diagnosed samples. The proposed approach encompasses distinctive technical attributes in a clinical scenario, as its minimal sample volume requirements and rapid results are compatible with a few drops of heel stick blood samples from newborns admitted to the neonatal intensive care unit. This would enable monitoring of both sepsis biomarkers within the initial hours after symptoms manifestation in high-risk neonates as a valuable tool in facilitating prompt and well-informed decisions about initiating antibiotic therapy. This is a clear example of the added value of micromotor technology for analyzing neonatal sepsis over time, opening new avenues in diagnostics based on low sample volumes. Additionally, it is demonstrated that a small sample volume and bubble generation, especially from hydrogen peroxide, do not affect biocompatibility or analyte detection efficiency. This accounts for the excellent agreement between the results obtained with this strategy and those obtained with the gold standard method used in hospitals. In general, it can be said that although the fuel concentration and the presence of surfactants can obviously affect the biological material, the key to this success is the low analysis times employed, which are compatible with the overall stability of the functionalized micromotor.^41^ Graphene QDs/Pt^42^ or Au/Pt catalytic tubular micromotors^43^ have been modified with fluorescently labeled DNA for miRNA and target DNA fluorescence detection, reaching LODs in the nanometer range. Reprimo, a highly glycosylated protein used as a gastric cancer biomarker, has also been detected with DNA-modified graphene/Pt/Ni tubular micromotors.^44^ Fuel-free magnetic micromotors have also been introduced to enhance the biocompatibility of catalytic micromotors for cell and bacteria aptassays. Magnetic spirulina has been used as a template for assembling an aptamer that is specific to , QDs as signal reporters, and polydopamine as the NIR-activated polymer for bacterial inactivation.^41,45^ Regarding the use of affinity peptides, tubular catalytic micromotors with outer carbon nanomaterials or 2D nanomaterial layers (WS_2_, MoS_2_, graphene)^28,46–48^ have been used for bacterial-related endotoxin detection. Figure 3B shows a strategy involving catalytic WS_2_/Pt and MoS_2_/Pt micromotors that are modified with a rhodamine-labeled affinity peptide. This strategy demonstrated excellent selectivity for the fast (60 s) and sensitive (1.9 ng mL^–1^) detection of endotoxin.^49,50^

Molecularly imprinted micromotors (MIMs) are an interesting alternative to sophisticated probes because they have tailored recognition abilities. Figure 3C shows a schematic of MIMs micromotors for α-bungarotoxin detection. Time-lapse images clearly illustrate the changes in the fluorescence emission on the micromotor surface (OFF in the absence of the fluorescent toxin, and ON in its presence), allowing for quantitative analysis.^29^ The MIM strategy has also been used in fuel-free, magnetically propelled designs to enhance biocompatibility, as illustrated for phycocyanin detection.^51^

In summary, fluorescence biosensing with micromotors shows great potential for detecting relevant biomarkers involving complex analytes, such as proteins and bacteria, using ultralow sample volumes. This is particularly important in cases involving neonates. Micromotor fluorescence-based approaches allow for direct, real-time detection with high sensitivity and selectivity thanks to a variety of fluorescent nanomaterials, tags, and receptors. Interestingly, these approaches can also be used in microplate readers for high-throughput analysis in a free-microscope format. However, more sample quantity is required in this case compared to elegant on the fly assays using fluorescence microscopy.

### Micromotor-Based Colorimetric (Bio)­sensing

As with other analytical technologies, approaches that allow for naked-eye detection are very appealing, because they are simple and inexpensive and can be integrated into portable strips. Representative examples of micromotors have illustrated a successful approach to cortisol detection using catalytic PEDOT micromotors that are modified with specific antibodies in connection with 3,3′,5,5′-tetramethylbenzidine (TMB), which allows for naked-eye detection.^52^ In the environmental realm, MnO_2_-based catalytic micromotors have been used to detect phenylenediamine isomers through the motion-induced generation of the corresponding colored isomers due to the generation of OH from the decomposition of the hydrogen peroxide fuel by MnO_2_, which is also used for catalytic propulsion. This reveals the dual role of MnO_2_ as catalyst and precursor agent for analyte detection.^53^ Janus Au-silica-Pt micromotors have been shown to enhance pepsinogen detection in lateral flow immunoassays. The motion of the micromotors modified with the specific receptors enhances fluid mixing and interactions with target analytes, increasing the sensitivity and also reducing the detection times in this assay format.^54^ Tubular chitosan/Prussian blue (PB) catalytic micromotors can be used for acetylthiocholinesterase (ATChE) encapsulation. As shown in Figure 4A, the micromotors were used in an inhibition assay format to detect neostigmine as a nerve agent. This analyte inhibits the enzyme’s action, preventing the poisoning of the PB catalytic layer and consequently allowing the oxidation of TMB by PB to produce a blue color. PB is also used as a catalyst for hydrogen peroxide for propulsion toward the oxygen bubble generation. Readout can be easily performed in a microplate reader, yet naked-eye detection is also possible.^55^

**Figure 4:**
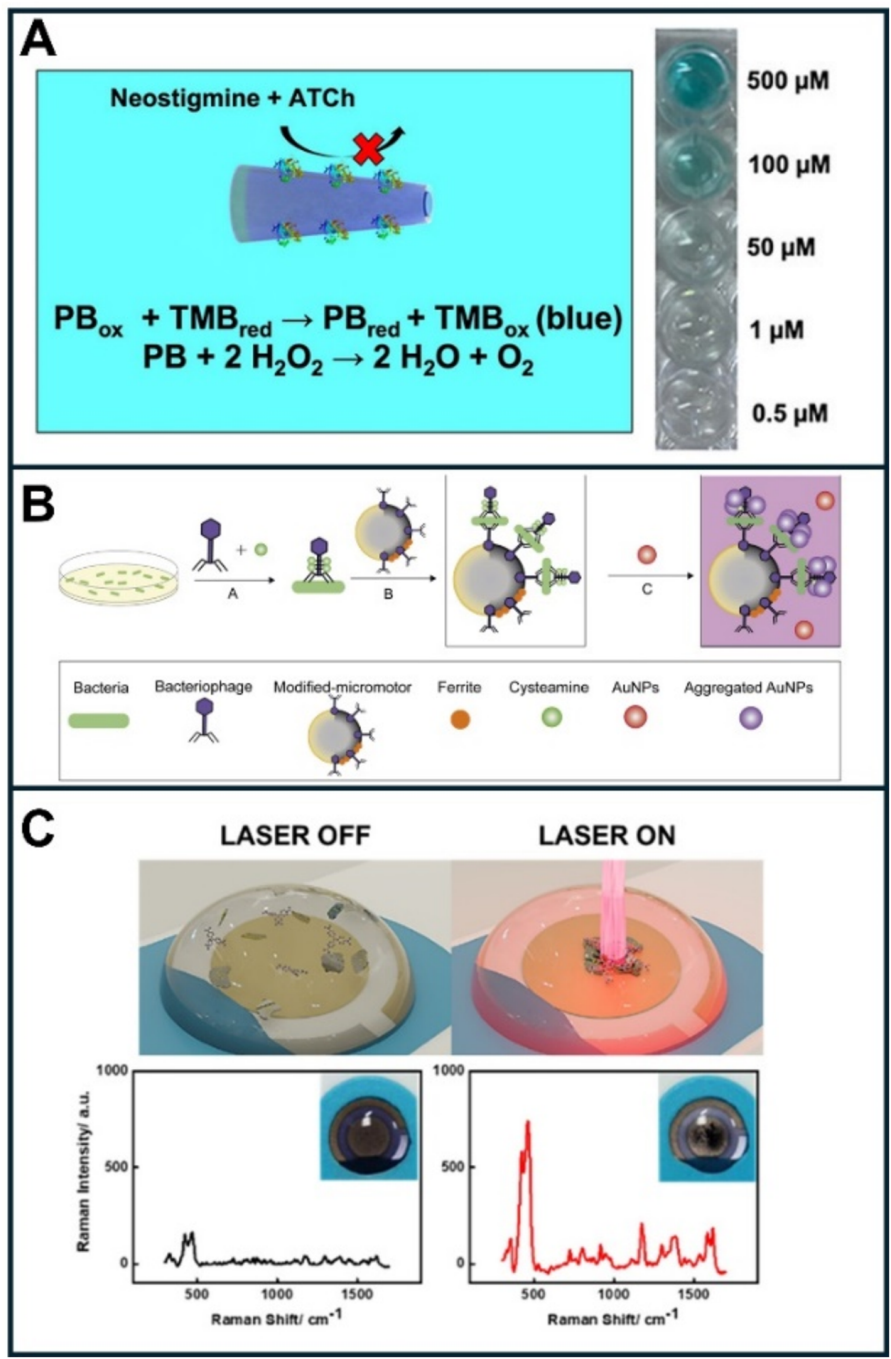
Optical and SERS (bio)­sensing using micromotors. (A) PB/chitosan micromotors encapsulating ATChE for colorimetric neostigmine detection. The assay’s principle is based on ATChE inhibition by neostigmine and colorimetric detection via TMB oxidation by PB, as illustrated in the figure’s photographs. (B) Strategy for detection using T4-bacteriophage-modified micromotors. (C) MoS_2_@Au photophoretic micromotors for surface-enhanced Raman scattering (SERS) sensing enhancement. When irradiated by the equipment’s laser, the micromotors exhibit schooling behavior, which concentrates the analyte and greatly enhances the signal (see the Raman spectra and time-lapse images on the bottom right part). Reprinted from ref 55 (A), ref 56 (B), and ref 57 (C).

To achieve exquisite selectivity and full biocompatibility, a magnetic micromotor-based assay using bacteriophage-modified micromotors in conjunction with gold nanoparticles has been integrated into microplate readers to enable the highly selective visual detection of bacteria. Graphene/ferrite Janus micromotors modified with a T4-bacteriophage are highly specific for capturing and detecting bacteria. The bacterium–thiolate phage complex is captured by the phage-modified micromotors. A gold nanoparticle solution is used as a probe for the colorimetric readout. See Figure 4B, for a detailed explanation of the strategy. A high bacterial concentration decreases the absorbance of AuNPs due to their higher aggregation by the action of cysteamine. The versatility and high selectivity of the protocol are illustrated by the analysis of urine and serum samples from hospital samples, which shows high agreement.^56^

The advantages of colorimetric detection, which allow rapid, visual, and on-site naked-eye detection, often without sophisticated instrumentation, have also proven to be very useful for micromotors. This is possible both directly, through color change by chemical reaction, and indirectly, through more sophisticated detection systems based on monitoring color change due to nanomaterial (AuNPs) size change. Thus, it can be said that this detection principle has not escaped micromotor technology, as expected.

### SERS (Bio)­sensing with Micromotors

Micromotors have also been elegantly applied as on-the-fly SERS substrates for sensing approaches. This concept was demonstrated using catalytic Au/SiO/Ti/Ag rolled-up engines for rhodamine 6G capture and signal enhancement.^58^ To avoid the use of peroxide fuel for further biocompatibility, magnetic rolled-up Au/SiO/Fe micromotors were explored.^59^ However, an additional magnetic generation setup is needed for propulsion. Interestingly, light-driven micromotors can solve this issue, as they can be propelled by the same source as the Raman equipment. Phototactic Ag@SiO_2_ micromotors can experience self-diffusiophoresis under UV light irradiation, quickly aggregating to enhance the SERS signal for crystal violet and MCF-7 cancer cell detection.^60^ Photophoretic MoS_2_@Au micromotors can also self-aggregate after irradiation with the equipment’s laser source, simultaneously allowing analyte preconcentration and enhancing the SERS signal, for the detection of malachite green, crystal violet, and paraquat, resulting in an impressive swarming of micromotors at speeds up to 5 mm/s (for more details, see Figure 4C).^57^ This unique collective on-the-move-based SERS strategy shows great promise for on-site detection with portable instrumentation in a myriad of environmental monitoring and clinical applications.

## Micromotor-Based Electrochemical (Bio)sensing

Electrochemical sensing is particularly attractive due to its inherent miniaturization and even portability. This makes it highly compatible with low sample volumes without compromising analytical performance. Consequently, it is also highly pertinent for coupling with micromotor technology, enabling creative bioassays on tailored electrode design.

While motion- and fluorescence-based detection can be performed on the fly, specific issues need to be considered when using electrochemical detection coupled with micromotor technology. Typically, micromotor-based bioassays based on electrochemical readout require a custom-designed external electrode. In this case, due to their magnetic properties, micromotors are retained on the electrode surface, and the analyte is monitored by diffusion to the electrode surface. In this format, antibody-modified carbon-based magnetic micromotors have been used to detect CRP protein in newborn samples for the diagnosis of possible sepsis infection, enabling fast detection using microliters of sample. The micromotors were modified with the specific antibody and then labeled with HRP-tagged secondary antibodies to facilitate electrochemical detection on external carbon screen-printed electrodes.^41^

Ideally, however, micromotor-based bioassays and electrochemical detection should be coupled online. As illustrated in Figure 5A, the antibody-modified carbon-based micromotor for assessing CRP was easily incorporated into a electrochemical microfluidic detection chip. As can be seen, the immunoassay and recognition were performed on the fly in a separate reservoir. The motion then stops due to the micromotor magnetic properties, after which the resulting immunocomplex is pumped for flow through amperometric enzyme-labeled protein detection. This avoids any potential crosstalk between analyte detection and the micromotors. Using this format, preterm neonatal samples were analyzed, again showing excellent agreement with hospital samples.^61^

**Figure 5:**
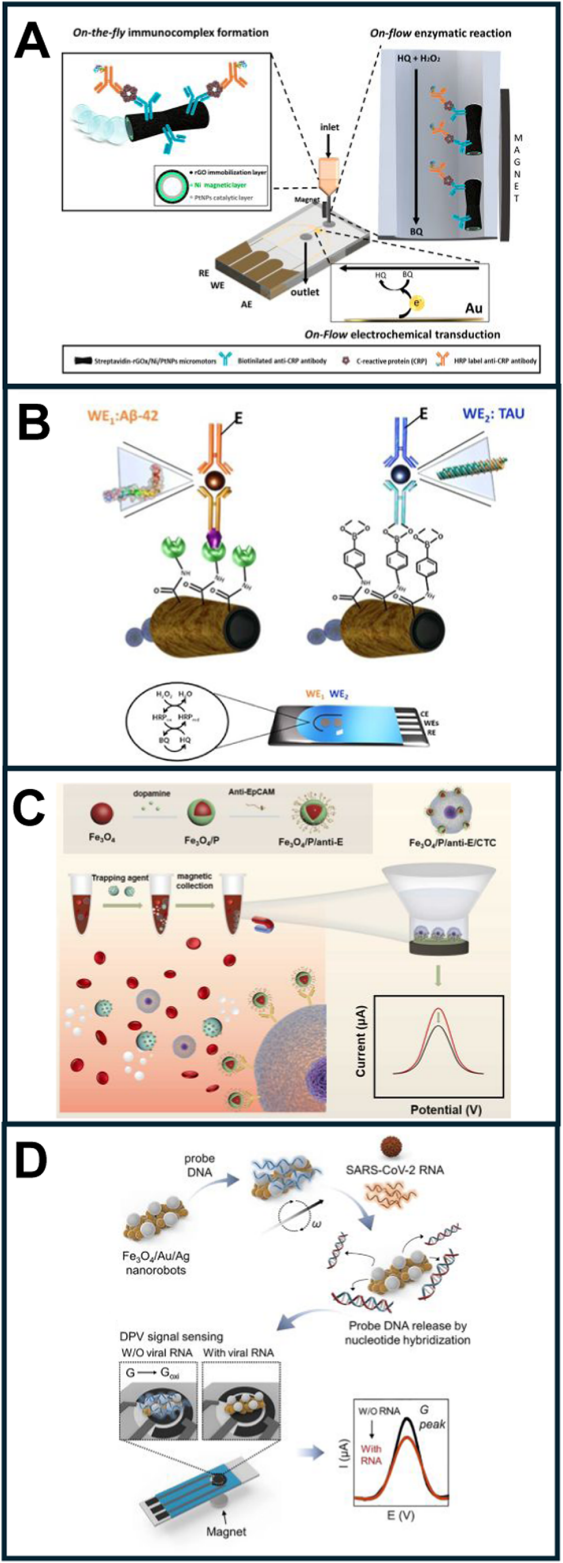
Electrochemical (bio)­sensing using micromotors. (A) Catalytic micromotor-based detection of CRP in an electrochemical microfluidic system. (B) A dual electrochemical micromotor-based assay for the simultaneous detection of Aβ-42 and Tau protein in brain, cerebrospinal fluid, and plasma samples from patients. The micromotors are modified with either streptavidin or aminophenylboronic acid to immobilize the antibodies, after which target capture and labeling with an HRP-tagged secondary antibody takes place. This allows for electrochemical detection on a dual screen-printed electrode using hydroquinone as a mediator. (C) Mg-based micromotors modified with antibodies for circulating tumor cell (CTC) detection in whole blood. (D) Magnetic micromotors modified with DNA for viral RNA detection. Reprinted from ref 61 (A), ref 62 (B) copyright 2025 Elsevier, ref 63 (C) copyright 2023 Elsevier, and ref 64 (D) copyright 2022 Elsevier.

Returning to the approaches in which micromotor-based bioassays and the electrochemical detection are carried out separately, the diagnosis of Alzheimer’s disease (AD) has recently also been explored using electrochemical immunoassays^65^ for amyloid β (Aβ-42) and aptassays^66^ for the related oligomer (AβO-42) on-board catalytic micromotors in small volumes of hard-to-obtain clinical samples of high significance, such as brain tissue, cerebrospinal fluid, and plasma. Excellent agreement with gold standard methods, low detection limits (in the pg/mL range), and a low sample volume required (5 μL) testify to the applicability of these approaches for fast, potential early Alzheimer’s disease diagnosis. Polypyrrole/nickel/platinum micromotors were used for determining Aβ-42 in a sandwich immunoassay, in which the micromotors were functionalized with a capture antibody and a secondary antibody with an electrochemical tag.^65^ An impedimetric aptasensor for AβO-42 was developed by using graphene/Pt/Ni micromotors that were modified with gold nanoparticles. These were used not only to covalently bind a specific thiolated-aptamer for the design of a label-free electrochemical sensor but also to improve the final micromotor propulsion performance due to their catalytic activity (approximately 2.0× speed). This on-the-move bioplatform provides a fast (5 min), and selective approach with an excellent LOD (0.10 pg/mL) and wide linear range of 0.5–500 pg/mL in ultralow volumes of a clinical samples from AD patients (5 μL), without any dilution.^66^ Recently, the simultaneous determination of Aβ-42 and Tau protein has also been demonstrated using a dual-electrode with different functionalization strategies for the capture antibody (streptavidin–biotin and boronic acid for Aβ-42 and Tau protein, respectively; see Figure 5B). Interestingly, no crosstalk was identified among the two micromotors - based bioassays performed in dual electrodes. Although the quantitative results are promising, the difficulty in measuring tau protein levels has been revealed.^62^

An interesting approach to true integration is to use one drop as an electrochemical cell in which micromotor-based bioassays and electrochemical detection are carried out simultaneously, resulting in a complete on-board electrode test. In this case, convection due to the self-vortex effect generated by the micromotors drives the mass transport of the analyte to the electrode, improving the efficiency of diffusion-controlled mass transport. Water-driven Mg micromotors have been explored for electrochemically assisted sensing in this format assay. The propulsion of Mg micromotors by the action of the supporting electrolyte via pitting corrosion and the associated OH generation and enhanced movement allow for the detection of nonelectroactive analytes all in one in screen-printed electrodes^67,68^ In the case of phthalates,^67^ the self-propulsion of the micromotors along the samples, according to Mg hydrolysis in water results in the generation of hydrogen bubbles for propulsion and hydroxyl ions for the degradation of diphenyl phthalate into phenol. This degradation product is electrochemically detected on site. This micromotor-based approach integrated all in one derivatization and detection and is an elegant example of complete, on-board, electrode micromotor-based electrochemical bioassay. When the cargo of reagents/analytes from the micromotor is released to the sensing spot, it may help mitigate potential crosstalk issues. The enhanced mass transport of Mg micromotors has also been exploited for glucose detection.^69^ Further exploration of off-line micromotor bioassays and electrochemical detection strategies has enabled the detection of important biomarkers in whole blood without previous treatment, paving the way for diagnoses using just one drop of blood. Oxidized low-density lipoprotein (LDL), a biomarker of atherosclerosis progression, has been detected in whole blood using Mg Fe_3_O_4_@PB micromotors modified with an antibody for Ox LDL. The micromotors spontaneously propel in the blood sample, enhancing the capture of the target. After dissolution of the Mg, the remaining magnetic shell with the captured analyte can be manipulated by a magnet toward a glassy carbon electrode for amperometric detection of the analyte.^70^ There is no crosstalk between the trapped metallic part of the micromotor and the electrode, which is an additional advantage of the approach. A similar configuration has been used to detect CTCs in cancer diagnosis^63^ and to detect α-synuclein as a biomarker for Parkinson’s disease.^71^ For example, Figure 5C depicts the strategy used to detect CTCs. Magnetic Fe_3_O_4_ nanoparticles modified with polydopamine and anti-EpCAM antibodies are embedded into Mg micromotors. The resulting micromotor moves through the entire blood sample, and the magnetic units interact with the CTCs. After Mg dissolution, the CTCs-magnetic unit complex is trapped in the electrode for electrochemical measurement.

Using a format in which the micromotor-based bioassay and the electrochemical detection are carried out separately, fuel-free Janus micromotor-based approaches have also been reported. Fuel-free magnetic-bead Janus micromotors modified with anti-SARS antibodies exhibit collective behavior to enhance capture and electrochemical detection via the hydrogen evolution reaction.^72^ A similar principle was employed for cancer cell detection^73^ and for the detection of viral RNA associated with SARS-CoV-2 using Fe_3_O_4_/Au/Ag micromotors modified with DNA. As seen in Figure 5D, moving micromotors interact with target RNA and release the target DNA–RNA complex. Guanine oxidation is measured by differential pulse voltammetry in screen-printed electrodes.

Overall, electrochemical (bio)­sensing strategies using micromotors are still under development. However, the promising results obtained in the analysis of relevant biomarkers in small sample volumes make these strategies particularly relevant in diagnostic settings. Due to the inherent miniaturization and versatility of electrochemical detection, micromotor technology can be readily adapted, coupled, and fully integrated on an electrode board with a myriad of ultraminiaturized electrodes of different sizes, shapes, and materials. Interestingly, this technology enables the entire bioassay to be carried out on the electrode surface, facilitating full coupling between the bioassay and on-site detection and even enabling multiplexed detection.

## Lab-on-a-Chip and Micromotors

Precise motion control is essential for meeting the demands of future lab-on-a-chip and microfluidic applications, where complex operations in relevant biomedical scenarios such as organ-on-a-chip are required. The precise control afforded by microfluidics facilitates the integration of organoids and cell cultures within the microchannels. This enables the unique and elegant use of micromotors for biosensing in these complex systems and opens up avenues in the field of drug delivery where precise biosensing is required. Micromotors’ unique ability to transport cargoes rapidly and in a controlled manner, coupled with their small size, makes them particularly attractive for (bio)­sensing and delivery in ultraminiaturized environments. Taking advantage of their remarkable ability to transport different loads, the combination of micromotors and lab-on-a-chip technology offers considerable potential for creating powerful autonomous ultraminiaturized analytical systems. Functionalized micromotors guided through microchip channels can pick up and transport target biomolecules to specific reservoirs for subsequent analysis. In this way, the common washing steps in bioaffinity assays can also be integrated. Magnetic guidance has become a useful and pertinent approach for micromotor motion and guidance through microchannel networks.^74–77^ Selected examples of this technology have been illustrated using PEDOT–COOH/Ni/Pt micromotors modified with immunoglobulin antibodies for the isolation of bacteria, targeting the protein A present in its cell wall. Labeling with a secondary antibody tagged with a microsphere allows for visualization of the capture event.^78^ Controlled movement and manipulation within microchip channels has also been successfully demonstrated.^79^ The concept has also been illustrated by capturing sugar-modified polystyrene beads using lectin-modified carbon-based micromotors^80^ or biotin-functionalized Janus micromotors that transport streptavidin-modified polyelectrolyte capsule, which trap charged molecules within microchip channels.^81^ Fuel-free models are also useful for biocompatible on-chip transport and operation, as they avoid the potential clogging of channels by oxygen bubbles from catalytic propulsion. For example, electric powered micromotors are able to dielectrophoretically capture and transport synthetic and biological (e.g., DNA, cells) cargoes within two reservoirs separated by a microchannel.^82,83^ The use of functionalized magnetic beads as cargo manipulated by electric and magnetic fields might be considered as a simplified approach, eliminates the need to functionalize the micromotor itself.^83^ Although the strategies are simplified, the range of commercially available beads is somewhat limited for determining certain analytes. For research purposes, particularly when using specific probes specifically designed for highly relevant analytes, micromotors offer great versatility and allow for tailored functionalization with no limitations on the type of material used (e.g., graphene, polymers, and so on).

## Cellular Sensing: a Bridge to *In Vivo* (Bio)sensing

The small size of micromotors, coupled with their targeted approach and delivery capabilities, holds considerable promise for extra- and intracellular (bio)­sensing approaches. Early proofs-of-concept applications have paved the way for the translation of micromotors potential for *in vivo* applications.^84^ A prerequisite is the use of biocompatible propulsion schemes. For example, urease-powered Janus micromotors in connection with DNA switches can detect pH changes in cellular environments,^85^ while ultrasound (US) propulsion using graphene-modified nanowires and fluorescence-labeled DNA probes is an effective approach for the intracellular detection of mRNA in cancer cells.^86,87^ The US propelled micromotors consisted of a Au nanowire coated with graphene oxide to serve as a support for π–π modification with a probe DNA labeled with QDs emitting at different wavelengths for multiplexed detection. Impressively, the micromotors permeate the cells and move under the action of ultrasound, releasing the probes for simultaneous quantitative detection of miR-10b and miR-21. While the previous intracellular sensing approaches seems promising, several challenges still need to be faced before *in vivo* biosensing can be achieved, ranging from potential biosafety issues and *in vivo* tracking of the micromotors to the evasion of the immune barrier.^88^ In addition, micromotors move fast only under conditions that are clearly not available *in vivo*, and in some cases, the analytical signals (electrochemical and optical) are not compatible with *in vivo* use. How can such challenges be solved? Recent works have illustrated the potential of certain micromotor designs for full biocompatible and autonomous motion in certain body compartments. In this context, Mg-based micromotors are particularly attractive for use in the mouth to treat bacterial infections, such as periodontitis^89^ and in the gastrointestinal track,^90^ due to their full biocompatibility. Magnetic micromotors also represent a convenient alternative when considering the possibility of controlled external operation using current magnetic resonance medical equipment. Optimal motion has been demonstrated in vitreous humor, peritoneal fluid, and blood.^91,92^ *In vivo* toxicity studies of silica-gold-coated helical micromotors have demonstrated the biocompatibility in mice at concentrations of up to 8 × 10^10^ nanomotors/kg body weight, which corresponds to 4.8 × 10^12^ nanomotors for an adult human weighing about 60 kg.^93^ However, it is unclear whether this will remain the case following functionalization with certain sensing probes and how stable they will be once they enter the human body. Another important issue that remains to be solved is the efficient tracking and localization of micromotors within the human body or a given organ.^94^ Ideally, this should be achievable using common biomedical imaging techniques, such as photoacoustic computer tomography, magnetic resonance imaging, or ultrasound tracking.^95^ To date, progress has been made in this direction, with the real-time visualization of micromotors achieved in mouse and rat models. This has been demonstrated in the tracking of micromotors in the intestines^96^ or the gastrointestinal track.^97^ In order to overcome these challenges and achieve simultaneous real-time imaging and detection with micromotors, gold nanorods and nanostars have been incorporated into the micromotors, primarily to improve contrast and specificity in connection with dual ultrasound and photoacoustic imaging.^98^ Such functionalization holds considerable promise for *in vivo* SERS sensing (facilitated by the gold layer), as well as for tracking the release of fluorescence drugs over time, allowing for continuous monitoring and *in vivo* biosensing,^99^ which remains to be demonstrated. Additionally, organ-on-chip technology is a very valuable tool for obtaining information for future *in vivo* biosensing due to its emulation of biological complexity. Microfluidics can indeed accurately replicate complex physiological conditions, including blood flow dynamics, tissue microarchitecture, and biochemical gradients.^100^ This capability enables the testing and optimization of micromotor navigation and functionality in environments that closely mimic *in vivo* conditions.

## Opportunities and Challenges

Micromotor technology has found applications in *in vitro* (bio)­sensing, mainly in the niche where samples are scarce or targeted detection is required to take place in well-defined, hard-to-reach localized (biological) areas. The field is clearly evolving from early speed-motion-based approaches to more sophisticated designs through the inclusion of specific receptors for highly sensitive and selective analyte detection. The type of micromotor, propulsion mechanisms, and materials used are tailored to improve the performance of the final (bio)­chemical sensing design and operation. Catalytic designs are extremely useful for performing bioassays in (extremely) low sample volumes due to the high towing force in combination with functionalization with specific receptors, resulting in an enhanced probe capture. However, fuel-free approaches are ideal for guided detections in well-defined, hard-to-reach localized biological areas while keeping the surrounding areas intact.

We can identify a plethora of micromotors, mostly catalytic, that are connected to detection principles and bioassay designs for analytes of high significance. In certain cases, these are used to analyze diagnosed clinical samples in relevant fields. The advantages of catalytic propulsion justify its widespread use in sensing: long-term operation (infinite, unless the fluid/drop evaporates), high towing force, materials versatility, and the ability to include magnetic layers for controlled motion and direction. It also simplifies washing steps within different assays. Fuel-free approaches using magnetic, ultrasound, or electric fields offer precise motion control and directionality as well as biocompatibility in (bio)­sensing, making them another asset of micromotor technology for targeted detection in hard-to-reach areas. The main drawbacks yet justified the few works available as compared with the progress in catalytic micromotors. Such disadvantages include a low towing force and reduced fluid mixing, resulting in poor operational performance.

Unfortunately, the analytical potential of micromotors for biosensing in complex diagnoses has been explored only in some real-world scenarios. Relevant examples include blood samples from neonates with very low birth weight, samples from Alzheimer’s patients at different stages of the disease, such as brain tissues and cerebrospinal fluid, and samples with low availability.

However, within this framework, micromotors have demonstrated excellent analytical performance in terms of selectivity and sensitivity with remarkable LODs. Progress so far is also promising, in line with some studies involving analytical validation using certified reference materials and even clinical samples, with excellent accuracy and correlations. Nevertheless, these analytical properties have rarely been demonstrated due to a lack of reference materials, and correlation studies involving diagnostic levels have been much less developed. Reported linear ranges that cover clinical ranges are also scarce. These are essential analytical metrics in the process of analytical validation of micromotor technology, which is still emerging in the field of (bio)­sensing. In all cases, the analysis time was short (1–15 min) and the sample volume was low (from 1 to 50 μL), both of which are valuable for diagnosis, especially in areas where access to clinical samples is difficult.

Consequently, more effort is needed in the analytical validation of the micromotor approaches involving diagnosed clinical samples with real complexity rather than just the analysis of spiked samples. New directions should mainly focus on developing multiplexed detection configurations that go beyond single analyte detection while maintaining low sample volumes. Indeed, many diagnoses are also time-dependent and require the sequential, multiplexed detection of several biomarkers to monitor disease progression, which increases the complexity of the diagnosis. This highlights the potential of micromotors for this purpose.

To name just a few relevant examples, future developments of (bio)­sensing-based micromotor approaches for other neonatal and neurodegenerative diseases, liquid biopsies for cancer diagnosis, dynamic collective cells, and bacteria detection as well as in other unexplored fields, such as forensic sciences (e.g., the detection of biomarkers in vitreous humor or crime scene evidence), are also immediately envisaged. The unique potential of micromotors to approach and penetrate cells for localized biomarker detection is also particularly attractive for vanguard diagnosis in organ-on-chip technology, opening the door to *in vivo* diagnosis. These key features are also extremely useful for overcoming some of the limitations of conventional biosensors.

The field of micromotors in (bio)­sensing can revolutionize modern (bio)­analysis even further by exploring new detection approaches that have not yet been investigated. Although micromotor integration into *smartphones* has been demonstrated, future translation into practical settings also lies in developing portable detection devices through strong multidisciplinary collaborations involving all clinical scenarios, with the aim of decentralized use by nonspecialized personnel. To this end, cross-disciplinary collaboration between scientists, medical doctors, and companies is undoubtedly needed to translate further potential into real-world applications, which will ultimately benefit the society.

In this exciting context of micromotor technology development, the door is beginning to open for *in vivo* analysis using micromotors, as reflected in the excellent recent literature. This will allow unprecedented advances in precision medicine, although it is still decades from practical applications. This requires closer collaboration between different disciplines, such as medicine, engineering, and science, as well as proper evaluation of biocompatibility and toxicity issues.^101–104^

In short, we humbly believe that micromotors have proven their relevance and potential in the field of disruptive *in vitro* (bio)­sensing ever since this technology was introduced two decades ago. Furthermore, we attempted to demonstrate the whole “collective” asset behind the micromotor-based bioassays. However, we believe even more strongly that micromotor technology still has much more to contribute to the next decade, given that it has a much more promising future than the present. This is our magical reality in which we work and believe in.
